# Editorial: Early-life environmental exposure and child development

**DOI:** 10.3389/fped.2023.1233644

**Published:** 2023-07-05

**Authors:** Hong Jiang, Fangbiao Tao, Mu Li

**Affiliations:** ^1^ School of Public Health, Key Laboratory of Health Technology Evaluation (National Health Commission), Fudan University, Shanghai, China; ^2^ School of Public Health, Anhui Medical University, Hefei, China; ^3^ MOE Key Laboratory of Population Health Across Life Cycle, Hefei, China; ^4^ School of Public Health, The University of Sydney, Sydney, NSW, Australia

**Keywords:** early life, environment, exposure, child, health, development

**Editorial on the Research Topic** Early-life environmental exposure and child development

Children's health is the foundation of the health and wellbeing of the entire population and is central to ensuring the sustainable development of society. Investing in children is one of the most critical investments for building a better future (1). In recent decades, child survival has been improved dramatically. The global under-five mortality rate fell from 93 deaths per 1,000 live birth in 1990 to 38 in 2021 (2). However, the WHO estimates that about 1.7 million children under five die from environment-related diseases every year worldwide (3), suggesting children's health is intricately linked to the environment. Exposure to adverse environmental factors in early life could have a short-term and long-term impact on children's health.

In this special issue, several Chinese studies examined exposure to environmental chemical pollutants in early life and on children's physical and cognitive development. Exposure to air pollution, especially indoor air pollution, has been found to be associated with an increased risk of childhood stunting (4). Yao et al. provided further observational evidence using the longitudinal data from the Chinese Family Panel Study between 2010 and 2018. Children living in households with solid fuel use had a higher risk of stunting over an 8-year follow-up. The risk was increased with types of solid fuel (firewood/straw) and prolonged exposure, while it was significantly decreased in children from households that had switched from solid fuels to clean fuels. The authors postulated that solid fuel use was a mediator of the relationship between poor socioeconomic factors (i.e., household income and parental education level) and childhood stunning. Based on a nationwide retrospective cohort study of 149,005 preschoolers in China, Wu et al. reported that about a quarter of children (23.9%) were exposed to second-hand smoking (SHS). In addition, the prevalence of suspected developmental coordination disorder was signiﬁcantly higher in the prenatal SHS-exposed group. Chlorpyrifos is an organophosphate insecticide. Products containing chlorpyrifos are used for pest control in agriculture for animal feed and food crops. In rural areas, children can be exposed to chlorpyrifos containing pesticides by eating contaminated food, breathing in, or getting them on the skin or in the eyes. In a cross-sectional study, Zhou et al. found higher levels of Chlorpyrifos exposure (determined by urinary chlorpyrifos levels) were associated with a higher risk of developing Attention-deficit/hyperactivity disorder in 1–6 years old Children from a rural area in Southwest China.

Early-life parental environment and nurturing care are essential to children's immediate health and lay the foundation of their lifelong health (5). Several papers focused on the importance of maternal health and wellbeing and parent's needs during pregnancy and postnatal. In a large Mother-Child Pairs Cohort study with 3,603 mother-child pairs, Shi et al. found children whose mothers were exposed to higher levels of psychological stress during pregnancy were more likely to exhibit poor neurodevelopment in infancy. However, sufficient responsive care in infancy diminished the relationships between stress in mothers during pregnancy and the neurodevelopment of children at 12 months of age. The most important factors for raising healthy children are knowledge and resources that parents and caregivers have to provide appropriate nurturing and care to children in early life. Cimen et al. demonstrated knowledge gaps among expectant parents in a lower-middle income country and their need for information such as children's essential development milestones and developmental risk indicators to monitor their growth and development. The paper also identified that fathers need to be involved in nurturing care to improve the family nurturing environment.

The studies in this special issue highlight that more high-quality research is needed to explore the relationships between environmental factors and child health and development. In the meantime, interventions such as parental education to address indoor pollution from solid fuel and second-hand smoking and access to clean fuels for low-income households should be implemented. Development of policies and programs supporting parents and caregivers to provide good care, adequate nutrition, and protection of children from any threats and harm, including environmental factors, so that they can reach their full genetic potential should also be the priority of governments.
